# New highlights of the syntheses of pyrrolo[1,2-*a*]quinoxalin-4-ones

**DOI:** 10.3762/bjoc.10.248

**Published:** 2014-10-14

**Authors:** Emilian Georgescu, Alina Nicolescu, Florentina Georgescu, Florina Teodorescu, Daniela Marinescu, Ana-Maria Macsim, Calin Deleanu

**Affiliations:** 1 Research Center Oltchim, Str. Uzinei 1, RO-240050, Ramnicu Valcea, Romania; 2 C. D. Nenitzescu Centre of Organic Chemistry, Romanian Academy, Spl. Independentei 202-B, RO-060023 Bucharest, Romania; 3 Petru Poni Institute of Macromolecular Chemistry, Romanian Academy, Aleea Grigore Ghica Voda 41-A, RO-700487 Iasi, Romania; 4 Research Dept., Teso Spec SRL, Str. Muncii 53, RO-915200 Fundulea, Calarasi, Romania

**Keywords:** 1,3-dipolar cycloadditions, multicomponent, one-pot three-component reactions, pyrrolo[1,2-*a*]benzimidazole, pyrrolo[1,2-*a*]quinoxalin-4-one

## Abstract

The one-pot three-component reactions of 1-substituted benzimidazoles with ethyl bromoacetate and electron-deficient alkynes, in 1,2-epoxybutane, gave a variety of pyrrolo[1,2-*a*]quinoxalin-4-ones and pyrrolo[1,2-*a*]benzimidazoles. The influence of experimental conditions on the course of reaction was investigated. A novel synthetic pathway starting from benzimidazoles unsubstituted at the five membered ring, alkyl bromoacetates and non-symmetrical electron-deficient alkynes in the molar ratio of 1:2:1, in 1,2-epoxybutane at reflux temperature, led directly to pyrrolo[1,2-*a*]quinoxalin-4-ones in fair yield by an one-pot three-component reaction.

## Introduction

The pyrrolo[1,2-*a*]quinoxaline system has significant biological activities and is a subject fo constant interest. This skeleton is a constituent of several bioactive heterocyclic compounds that demonstrate interesting activity against *Mycobacterium tuberculosis* [1], anti-HIV [2], anticancer [3], and it modulates the estrogen receptor activity [4].

Synthetic methods towards pyrrolo[1,2-*a*]quinoxaline derivatives based on pyrroles [5], or quinoxalines [6] have been recently reviewed. Among other synthetic routes, the 1,3-dipolar cycloaddition of heterocyclic *N*-ylides with various activated alkynes or alkenes is an important method for constructing fused heterocyclic systems such as pyrrolo[1,2-*a*]quinoxaline and pyrrolo[1,2-*a*]benzimidazole [7–13]. The development of more efficient synthetic methods towards these compounds is an active research area [14–16].

Recently, we reported on the formation of pyrrolo[1,2-*a*]benzimidazoles along with pyrrolo[1,2-*a*]quinoxalines in the one-pot three-component reaction of 1-benzylbenzimidazoles, phenacyl bromides and non-symmetrical activated alkynes in presence of propenoxide or 1,2-epoxybutane used as acid scavenger and reaction solvent [16]. These results prompted us to further investigate 1,3-cycloaddition reactions of 1-substituted 3-(alkoxycarbonylmethyl)benzimidazolium ylides with various dipolarophiles under the same reaction conditions, aiming to explore the generality of the reaction.

The previously reported data on 1,3-cycloaddition reactions of 1-substituted 3-(alkoxycarbonylmethyl)benzimidazolium ylides with various dipolarophiles are rather contradictory. Thus, 1-alkyl-3-(methoxycarbonylmethyl)benzimidazolium bromides with dimethyl acetylenedicarboxylate (DMAD) in presence of K_2_CO_3_ in DMF [7] or in presence of triethylamine in acetonitrile [8] give a mixture of pyrrolo[1,2-*a*]benzimidazole (2–7%) and a compound whose formation involves the loss of an alcohol molecule for which different structures have been proposed [7–8]. The correct structure of 2,3-dicarbomethoxy-5-methylpyrrolo[1,2-*a*]quinoxalin-4-one and the reaction mechanism was proposed by Meth-Cohn [9].

The reactions of 1-substituted 3-(ethoxycarbonylmethyl)benzimidazolium bromides with fluoroalkenes [10] or fluorovinyl tosylates [11] in presence of K_2_CO_3_ and triethylamine in DMF at 70 °C, or with activated alkenes, such as acrylates, acrylonitrile or diethyl malonate, in presence of triethylamine and an oxidant in DMF at 90 °C, led only to the normal cycloaddition products, i.e., pyrrolo[1,2-*a*]benzimidazoles [12]. When the same reactions were performed with polarized alkenes, such as 2-ethoxy acrylonitrile or 1,1-bis(methylthio)-2-nitroethylene, in presence of K_2_CO_3_ in chloroform at room temperature, only pyrrolo[1,2-*a*]quinoxalin-4-ones resulted in fair yields [13].

Our interest in developing simple synthetic pathways towards *N*-bridged heterocyclic compounds [17–20] prompted us to investigate the one-pot three-component reactions of various substituted benzimidazoles with alkyl bromoacetates and electron-deficient alkynes in presence of an epoxide. Herein, we report a simple one-pot three-component synthetic procedure towards pyrrolo[1,2-*a*]quinoxalin-4-ones and pyrrolo[1,2-*a*]benzimidazoles and we describe the influence of reaction conditions on the ratio of the two final reaction products. We developed also a selective one-pot three-component synthetic pathway towards pyrrolo[1,2-*a*]quinoxalin-4-one derivatives starting from benzimidazole derivatives unsubstituted at the five membered ring, alkyl bromoacetates and non-symmetrical electron-deficient alkynes in the molar ratio of 1:2:1, in 1,2-epoxybutane at reflux temperature.

## Results and Discussion

**Scheme 1:**
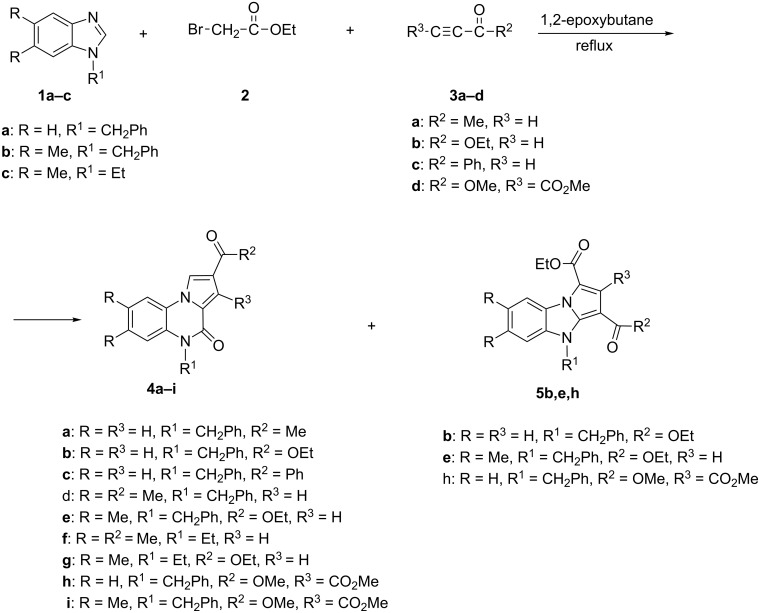
Synthesis of pyrrolo[1,2-*a*]quinoxalin-4-ones **4** and pyrrolo[1,2-*a*]benzimidazoles **5**.

The one-pot three-component reaction of 1-substituted benzimidazoles **1a–c**, ethyl bromoacetate **2** and non-symmetrical activated alkynes **3a–c**, in almost equimolar amounts, performed in presence of 1,2-epoxybutane gave pyrrolo[1,2-*a*]quinoxaline-4-ones **4a–g** as major reaction products. Pyrrolo[1,2-*a*]benzimidazoles **5b**,**e** were isolated along with pyrrolo[1,2-*a*]quinoxaline-4-ones **4b**,**e** only in some cases (Scheme 1, Table 1). All reactions have been performed by mixing the starting components at room temperature in 1,2-epoxybutane and heating the reaction mixture for 24 hours at reflux temperature. Pyrrolo[1,2-*a*]quinoxalin-4-one derivatives **4** were isolated from the reaction mixture by crystallization. To separate pyrrolo[1,2-*a*]benzimidazole derivatives **5**, each filtrate was concentrated under vacuum and chromatographed on a SiO_2_ packed column.

The HPLC analysis of crude reaction products indicated that small amounts of pyrrolo[1,2-*a*]benzimidazoles **5** were formed in all reactions, but they could not be always isolated from the reaction mixtures.

Due to the high reactivity of dimethyl acetylenedicarboxylate which can react also with the starting 1-substituted benzimidazole, the one-pot three-component synthetic procedure starting from almost equimolar amounts of 1-substituted benzimidazole **1**, ethyl bromoacetate and dimethyl acetylenedicarboxylate (**3d**) in 1,2-epoxybutane led to a complex mixture of reaction products. However, by direct reaction of 1-benzyl-3-ethoxycarbonylmethylbenzimidazolium bromide, obtained previously from 1-benzylbenzimidazole (**1a**) and ethyl bromoacetate (**2**), with dimethyl acetylenedicarboxylate (**3d**), in 1,2-epoxybutane at reflux temperature, the pyrrolo[1,2-*a*]quinoxalin-4-one (**4h**) was obtained as major reaction product along with a small amount of pyrrolo[1,2-*a*]benzimidazole (**5h**). Starting from 1-benzyl-5,6-dimethyl-3-ethoxycarbonylmethylbenzimidazolium bromide and dimethyl acetylenedicarboxylate **3d**, in the same conditions, only pyrrolo[1,2-*a*]quinoxalin-4-one **4i** was isolated from the reaction mixture (Scheme 1).

The yields and melting points of newly synthesized pyrrolo[1,2-*a*]quinoxalin-4-ones **4** and pyrrolo[1,2-*a*]bezimidazoles **5** are presented in Table 1.

**Table 1:** Synthesized pyrrolo[1,2-*a*]quinoxalin-4-ones **4** and pyrrolo[1,2-*a*]bezimidazoles **5**.

Entry	Reaction products					

	**4**	mp (°C)	Yield (%)^a^	**5**	mp (°C)	Yield (%)^a^

1	**4a**	225–227	39	–	–	–
2	**4b**	178–180	42	**5b**	130–132	13
3	**4c**	220–222	57	–	–	–
4	**4d**	274–275	43	–	–	–
5	**4e**	215–217	38	**5e**	191–193	21
6	**4f**	283–285	48	–	–	–
7	**4g**	191–193	39	–	–	–
8	**4h**	259–261 258–259 [8]	42 12 [8]	**5h**	177–178	16
9	**4i**	275–276	19	–	–	–

^a^Yields for isolated and purified compounds.

**Scheme 2:**
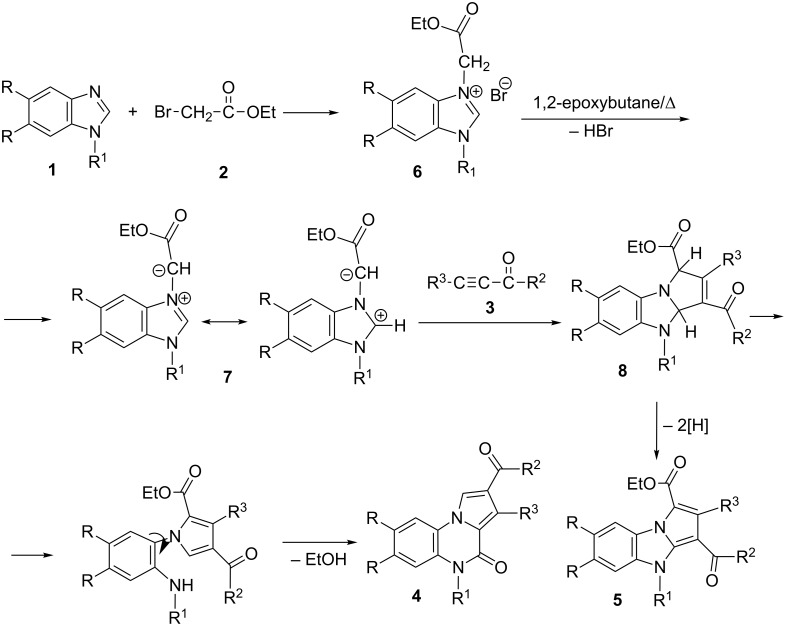
Reaction pathway leading to the formation of pyrrolo[1,2-*a*]quinoxalin-4-ones **4** and pyrrolo[1,2-*a*]bezimidazoles **5**.

The reaction pathway (Scheme 2) involves the quaternization of 1-substituted benzimidazoles **1** with ethyl bromoacetate (**2**) leading to corresponding benzimidazolium bromides **6**. The attack of the bromine ion from the benzimidazolium bromide on the oxirane ring in 1,2-epoxybutane results in ring opening and generation of the benzimidazolium *N*-ylide **7** by the action of the alkoxide. The benzimidazolium *N*-ylide **7** reacts with the activated alkynes **3** to give the corresponding primary cycloadduct dihydropyrrolo[1,2-*a*]benzimidazoles **8**. The formation of pyrrolo[1,2-*a*]quinoxalin-4-ones **4** involves the imidazole ring-opening, initiated by the deprotonation at C-1 of the primary cycloadducts **8**, followed by ring-closure involving the carbethoxy C=O group, a previously proposed rationale [9]. The formation of pyrrolo[1,2-*a*]benzimidazoles **5** involves the spontaneous in situ dehydrogenation of the primary cycloadducts **8**.

In order to explain the above mentioned results, we investigated the influence of reaction conditions on the ratio of the final reaction products **4** and **5** in 1,3-dipolar cycloaddition reactions of the 1-benzyl-3-(ethoxycarbonylmethyl)benzimidazolium bromide **6** (R = H, R^1^ = benzyl) with ethyl propiolate (**3b**) and DMAD (**3d**), in different reaction conditions reported in literature (Table 2). In these experiments, all crude reaction products were treated with aqueous solution of 5% HCl and extracted with CHCl_3_. The chloroformic extracts were dried, concentrated under vacuum, analyzed by HPLC and the peak areas of the final reaction products **4** to **5** were determined (Table 2).

**Table 2:** The influence of the reaction conditions on the final reaction products.

Entry	Reaction conditions	Ratio of peak areas^a^	

		**4b:5b**	**4h:5h**

1	1,2-epoxybutane, 24 h at reflux temperature (≈62 °C)	7.6	6.2
2	NEt_3_ and TPCD in DMF, 4 h at 90 °C^b^	7.7	2.7
3	NEt_3_ in acetonitrile, 4 h at reflux temperature (≈80 °C)^c^	46	54
4	K_2_CO_3_ in DMF, 48 h at rt^d^	27	–
5	K_2_CO_3_ + NEt_3_ in DMF, 24 h at 70 °C^e^	91	–

^a^Calculated from HPLC chromatograms; ^b^reaction conditions according to [12]; ^c^according to [8]; ^d^according to [7]; ^e^according to [10–11].

The results suggest that in the presence of an organic and/or inorganic base the formation of pyrrolo[1,2-*a*]quinoxalin-4-one derivatives **4** is favored, while in a neutral medium or in the presence of oxidants, such as TPCD [12], significant quantities of pyrrolo[1,2-*a*]benzimidazoles **5**, the normal 1,3-dipoar cycladdition product, are also formed. In this way, the low yields of pyrrolo[1,2-*a*]benzimidazoles **5** reported in literature [7–8] can be explained.

**Scheme 3:**
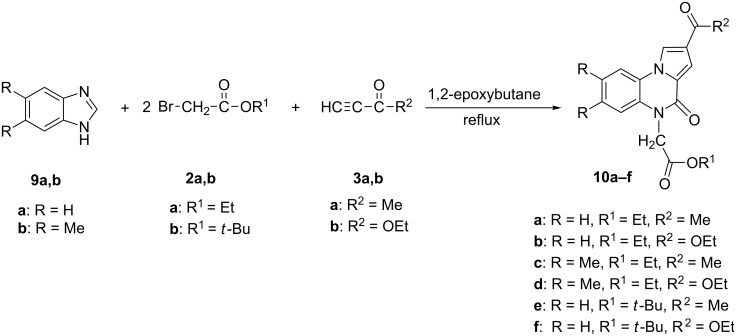
Novel synthetic pathway towards pyrrolo[1,2-*a*]quinoxalin-4-ones **10**.

An easy access to pyrrolo[1,2-*a*]quinoxalin-4-ones **10** was provided by the one-pot three-components reaction of benzimidazoles unsubstituted at the imidazole ring **9a**,**b** with alkyl bromoacetates **2a**,**b** and non-symmetrical, electron-deficient alkynes **3a**,**b**, in the molar ratio 1:2:1, in 1,2-epoxybutane at reflux temperature. This novel synthetic procedure lead directly to pyrrolo[1,2-*a*]quinoxalin-4-ones **10a–f**, as solely reaction product, in fair yields (Scheme 3).

A range of benzimidazole, unsubstituted at the imidazole ring and bearing various substituents on the benzoanelated ring, could be used as starting compounds. The reaction could be extended for a broad range of alkyl bromoacetates and non-symmetrical electron-deficient alkynes. Mild reaction conditions are involved, implying mixing the benzimidazole derivative with an alkyl bromoacetate and a non-symmetrical activated alkyne in the molar ratio of 1:2:1 at room temperature in 1,2-epoxybutane, then heating the reaction mixture at reflux temperature for 30 hours. All final pyrrolo[1,2-*a*]quinoxalin-4-one compounds have been isolated by simple, non-chromatographic methods.

The reaction pathway involves the intermediate *N*-alkylation of the imidazole ring with one equivalent of alkyl bromoacetate yielding 1-ethoxycarbonylmethylbenzimidazole, followed by its quaternization with the second equivalent of alkyl bromoacetate leading to 1,3-di(ethoxycarbonylmethyl)benzimidazolium bromide. The final pyrrolo[1,2-*a*]quinoxalin-4-ones are obtained according to the mechanism presented in Scheme 2.

The structures of newly synthesized pyrrolo[1,2-*a*]quinoxalin-4-ones **4** and **10**, and pyrrolo[1,2-*a*]benzimidazoles **5** were assigned by elemental analysis, IR and NMR spectroscopy. The ^1^H, ^13^C and ^15^N NMR chemical shifts have been unambiguously assigned based on the following 2D NMR experiments: H,H-COSY, H,C-HSQC, H,C-HMBC, H,N-HMBC, H,H-NOESY.

**Figure 1:**
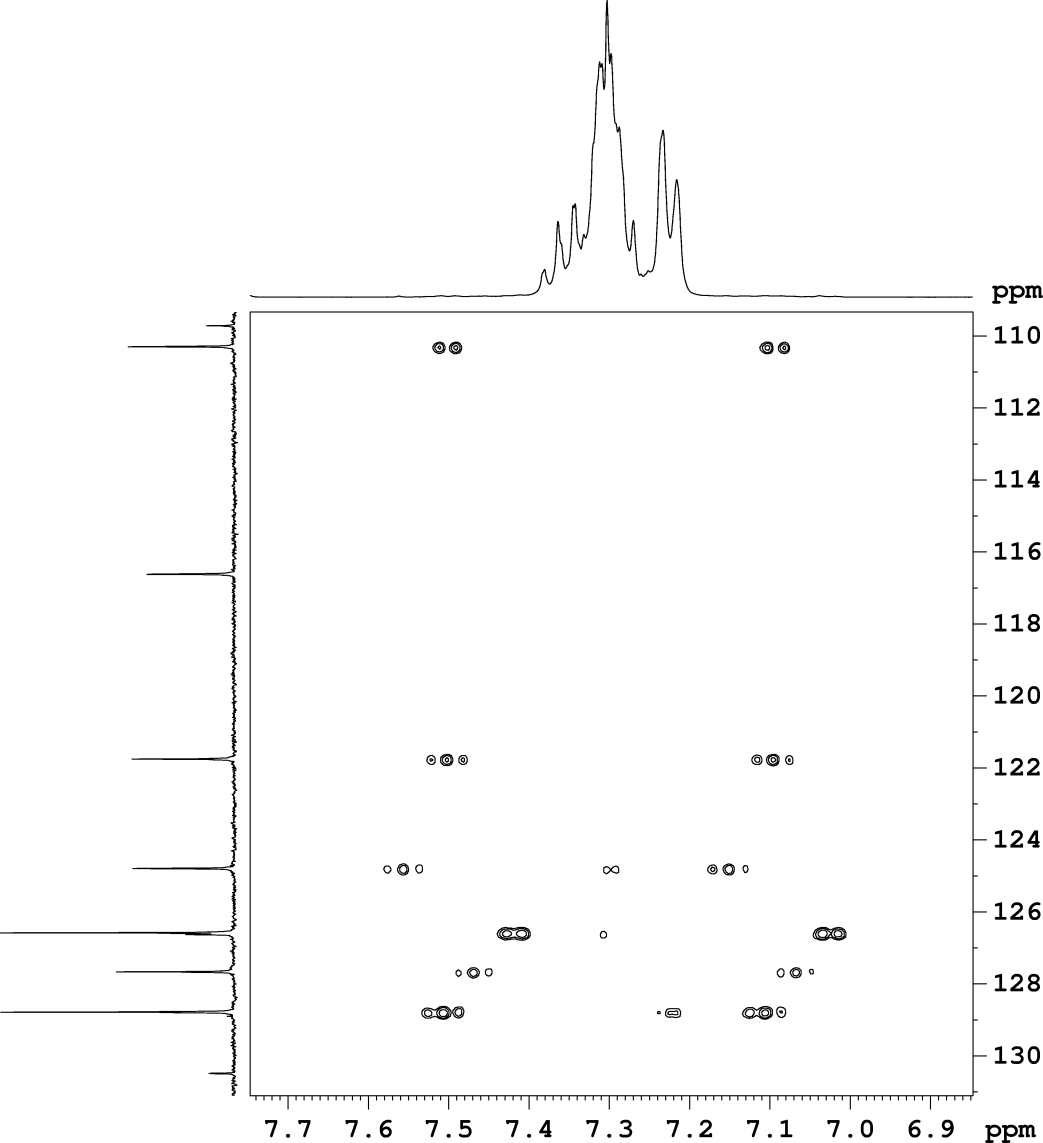
Undecoulpled H,C-HSQC spectrum for compound **5h**.

**Figure 2:**
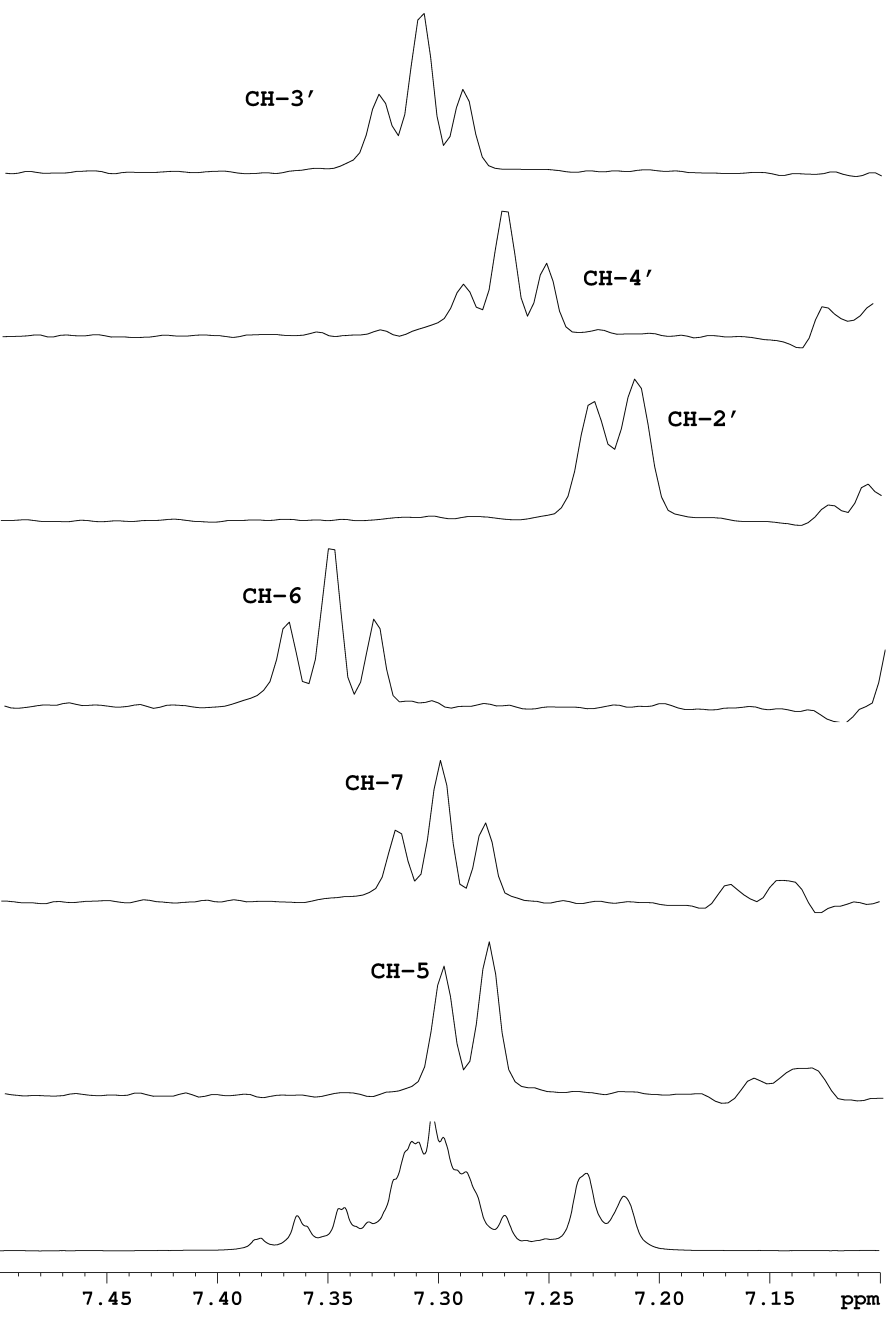
Individual ^1^H signal assignments based on ^13^C traces from H,C-undecoulpled-HSQC spectrum around the low field ^13^C satellite, in comparison with the ^1^H NMR spectrum (bottom) for compound **5h**.

In the ^1^H NMR spectra of pyrrolo[1,2-*a*]quinoxalines and pyrrolo[1,2-*a*]benzimidazoles the protons from the phenyl ring and the annelated benzo ring are overlapping in the region of 7–8 ppm. Based on a less used undecoupled H,C-HSQC type of spectrum we assigned for the first time the individual aromatic signals, the multiplicity and the order of magnitude of the coupling constants for these classes of compounds. The full assignments are listed in the experimental section and an example is shown in Figure 1 for compound **5h**. Thus, in Figure 1, one can clearly see separated cross peaks around each ^13^C satellite corresponding to all ^1^H signals in the region of 7.0–7.6 ppm. The low intensity ^13^C satellites in the ^1^H NMR spectrum are located outside (low and high field) of the region of the main ^1^H signal. When extracting 1D rows from the 2D H,C-undecoupled-HSQC spectrum corresponding to each ^13^C signal, one can see traces showing individual ^1^H signals (Figure 2). The pseudo 1D spectra from Figure 2 are traces at each ^13^C signal around the low field ^13^C satellite in the ^1^H dimension. In contrast with the normal ^1^H NMR spectrum (Figure 2, bottom) the pseudo 1D ^1^H spectra show individual signals allowing for the determination of chemical shifts, multiplicities, and coupling constants.

**Figure 3:**
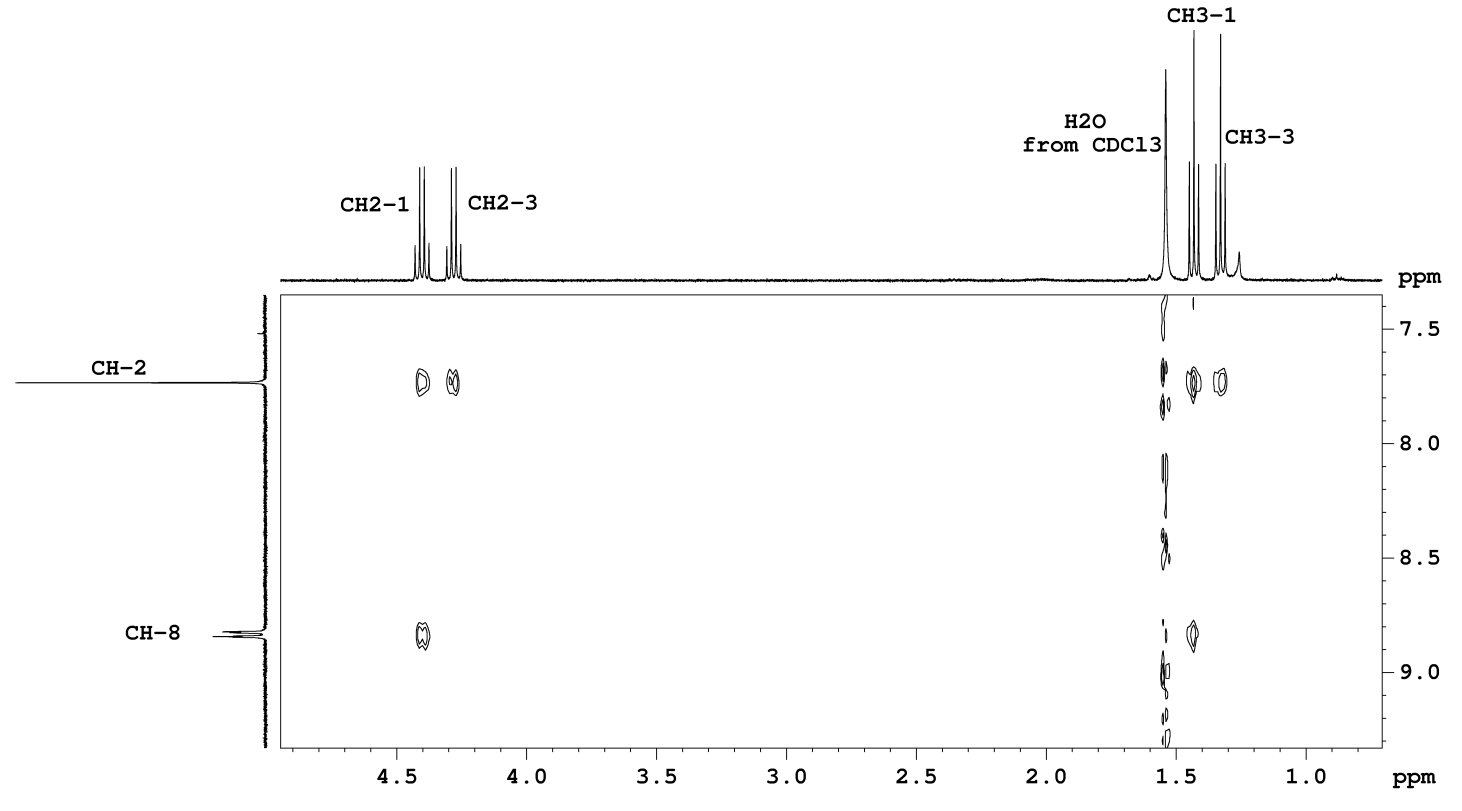
NOE response as cross peaks between carbethoxy group protons and protons from positions 2 and 8 of the heterocycle in a detail from the H,H-NOESY spectrum for compound **5b**.

For compounds **4h**, **4i**, **5b** and **5e**, the carbomethoxy respectively carbethoxy residues were assigned based on their NOE response. Thus, for compounds **4h**,**i** the methyl protons from carbomethoxy groups situated in positions 2 and 3 were differentiated based on their NOE cross peak with the proton in position 1. For compounds **5b**,**e** the protons from carbethoxy groups situated in positions 1 and 3 were assigned based on their NOE cross peaks with the proton in position 8, an example for **5b** is shown in Figure 3.

Based on the NOE assignments of various ethyl groups, we suppose that the preferred conformation in solution for the carbethoxy group in position 1 in compounds **5b**,**e**,**h** is oriented towards the benzo-annelated nucleus, thus the aromatic ring current inducing a deshielding effect on the CH_2_ and CH_3_ groups. On the contrary, in compounds **10a**–**d** we assume a solution preferred orientation of the carbethoxy group in position 5-N-CH_2_- of the heterocycle away from the benzo-annelated nucleus and on the same side with the carbonyl group, the latter inducing a shielding effect on the CH_3_ group.

## Conclusion

We have demonstrated that 1,3-dipolar cycloaddition reactions of 1-benzyl-3-(alkoxycarbonylmethyl)benzimidazolium ylides with activated alkynes led to a mixture of pyrrolo[1,2-*a*]quinoxalin-4-ones and pyrrolo[1,2-*a*]benzimidazoles. Pyrrolo[1,2-*a*]quinoxalin-4-ones are always the major reaction product and the ratio of pyrrolo[1,2-*a*]quinoxalin-4-one to pyrrolo[1,2-*a*]benzimidazole depends on reaction conditions and reactant structures.

A selective one-pot three-component synthetic protocol providing easy access to a wide range of pyrrolo[1,2-*a*]quinoxalin-4-one derivatives starts from benzimidazole unsubstituted at the imidazole ring, alkyl bromoacetates and non-symmetrical electron-deficient alkynes in the molar ratio 1:2:1, in 1,2-epoxybutane, enabling thus the expansion of studies on the biological properties of these compounds.

## Experimental

**General.** Melting points were measured on a Boëtius hot plate microscope and are uncorrected. The IR spectra were recorded on a Nicolet Impact 410 spectrometer, in KBr pellets. The high performance liquid chromatography (HPLC) analyses were performed with an Agilent Chromatograph 1200 Series at room temperature by isocratic elution of acrylonitrile on an Agilent Zorbax SB-C18 (250 × 4.6) column with a flow rate of 1.0 mL/min. The NMR spectra have been recorded on a Bruker Avance III 400 instrument operating at 400.1, 100.6 and 40.6 MHz for ^1^H, ^13^C, and ^15^N nuclei respectively. Samples were transferred in 5 mm Wilmad 507 NMR tubes and recorded with either a 5 mm multinuclear inverse detection z-gradient probe (^1^H spectra and all H,H/H,C/H,N 2D experiments) or with a 5 mm four nuclei direct detection z-gradient probe for ^13^C spectra. Chemical shifts are reported in δ units (ppm) and were referenced to internal TMS for ^1^H nuclei, to the internal deuterated solvent for ^13^C nuclei (CDCl_3_ referenced at 77.0 ppm), and referenced to liquid ammonia (0.0 ppm) using nitromethane (380.2 ppm) as external standard for ^15^N nuclei. Unambiguous 1D NMR signal assignments were made based on 2D NMR homo- and heteronuclearcorrelations. H,H-COSY, H,H-NOESY, H,C-HSQC and H,C-HMBC experiments were recorded using standard pulse sequences in the version with z-gradients, as delivered by Bruker with TopSpin 2.1 PL6 spectrometer control and processing software. H,C-undecoupled-HSQC experiments have been recorded using the pulse sequence described by S. Simova [21]. The ^15^N chemical shifts were obtained as projections from the 2D indirectly detected H,N-HMBC spectra, employing a standard pulse sequence in the version with z-gradients as delivered by Bruker (TopSpin 2.1 PL6). Elemental analyses for C, H and N were obtained using a COSTECH Instruments EAS32. Satisfactory microanalyses for all new compounds were obtained.

Benzimidazole, 5,6-dimethylbenzimidazole, activated acetylenic esters, 3-butyn-2-one and alkyl bromoacetates were purchased from Aldrich and used without further purification. 1-Benzylbenzimidazole, 1-benzyl-5,6-dimethylbenzimidazole and 1-ethyl-5,6-dimethylbenzimidazole were obtained from corresponding benzimidazoles and benzyl chloride, respectively ethyl bromide. 1-Benzylbenzimidazolium bromides (**6**) were obtained from 1-benzylbenzimidazole, respectively 1-benzyl-5,6-dimethylbenzimidazole, and alkyl bromoacetate in acetone, according previously reported methods [8]. Tetrapyridinecobalt(II) dichromate (TPCD) was obtained according the reported method [22].

**General procedure for the reaction of 1-substituted benzimidazoles (1a–c) with ethyl bromoacetate (2) and non-symmetrical alkynes (3a–c) in 1,2-epoxybutane.** A mixture of 1-substituted benzimidazole **1a–c** (2 mmol), ethyl bromoacetate **2** (2 mmol) and an alkyne **3a–c** (2 mmol) in 30 mL of 1,2-epoxybutane was heated at reflux temperature (approx. 62 °C) for 24 hours. The solvent was partly removed under vacuum, 3 mL of MeOH was added under a gentle stirring, and the mixture was left 2 hours in the refrigerator. The solid formed was filtered off and recrystallized from MeOH/Et_2_O giving pyrrolo[1,2-*a*]quinoxalin-4-one **4a–g**. The filtrate was concentrated under vacuum and chromatographed on a SiO_2_ packed column by eluting with EtOAc:hexane (1:4 v/v) giving pyrrolo[1,2-*a*]benzimidazole **5** and an additional quantity of pyrrolo[1,2-*a*]quinoxalin-4-one **4** (the order of elution: **4**<**5**).

**Ethyl 4-oxo-5-benzylpyrrolo[1,2-*a*]quinoxalin-2-carboxylate (4b).** 0.29 g (42%) pale yellow crystals. FTIR (ν_max_, cm^−1^): 3121, 2975, 1710, 1651, 1611, 1551, 1519, 1426, 1361, 1305, 1270, 1196, 1165, 1096, 1023; ^1^H NMR (CDCl_3_) δ 1.41 (3H, t, 7.2 Hz, CH_3_), 4.38 (2H, quartet, 7.2 Hz, CH_2_), 5.50 (2H, bs, CH_2_), 7.19–7.33 (8H, m, aromatic rings), 7.68 (1H, d, 1.6 Hz, H-3), 7.72–7.73 (1H, m, H-9), 8.24 (1H, d, 1.6 Hz, H-1). The individual chemical shifts, multiplicities and coupling constants for the 7.19–7.33 multiplet were obtained from undecoupled HSQC as follows: 7.21 (1H, m, H-8), 7.236 (1H, t, 8.1 Hz, H-7), 7.239 (1H, d, 8.2 Hz, H-6), 7.25 (1H, t, 7.4 Hz, H-4’), 7.28 (2H, d, 7.2 Hz, H-2’), 7.31 (2H, t, 7.3 Hz, H-3’) ppm; ^13^C NMR (CDCl_3_) δ 14.38 (CH_3_), 45.12 (CH_2_), 60.57 (OCH_2_), 113.97 (C-3), 114.99 (C-9), 116.88 (C-6), 119.42 (C-1), 120.43 (C-2), 123.27 (C-8), 123.37 (C-9a), 123.59 (C-3a), 126.58 (C-2’), 126.78 (C-7), 127.45 (C-4’), 128.87 (C-3’), 129.97 (C-5a), 136.04 (C-1’), 155.48 (C-4), 163.77 (COO); ^15^N NMR (CDCl_3_) δ 136.4 (N-5), 173.5 (N-10) ppm; anal. calcd for C_21_H_18_N_2_O_3_ (346.38): C, 72.82; H, 5.24; N, 8.09%; found: C, 72.90; H, 5.31; N, 8.01%.

**Diethyl 4-benzyl-4*H*-pyrrolo[1,2-*a*]benzimidazole-1,3-dicarboxylate (5b).** 0.1 g (13%) pale yellow crystals. FTIR (ν_max_, cm^−1^): 1700, 1685, 1580, 1514, 1479, 1453, 1400, 1303, 1290, 1233, 1181, 1136, 1106, 1070; ^1^H NMR (CDCl_3_) δ 1.37 (3H, t, 7.2 Hz, CH_3_-3), 1.48 (3H, t, 7.2 Hz, CH_3_-1), 4.32 (2H, quartet, 7.2 Hz, CH_2_-3), 4.45 (2H, quartet, 7.2 Hz, CH_2_-1), 6.13 (2H, bs, CH_2_), 7.25–7.32 (8H, m, aromatic rings), 7.78 (1H, s, H-2), 8.88 (1H, d, 8.2 Hz, H-8). The individual chemical shifts, multiplicities and coupling constants for the 7.25–7.32 multiplet were obtained from undecoupled HSQC as follows: 7.240 (2H, d, 7.5 Hz, H-2’), 7.248 (1H, t, 7.3 Hz, H-4’), 7.25 (1H, d, 8.2 Hz, H-5), 7.26 (1H, t, 8 Hz, H-7), 7.29 (2H, t, 7.4 Hz, H-3’), 7.30 (1H, t, 8 Hz, H-6) ppm; ^13^C NMR (CDCl_3_) δ 14.47 (CH_3_-3), 14.59 (CH_3_-1), 48.48 (CH_2_), 59.93 (CH_2_-3), 60.24 (CH_2_-1), 91.75 (C-3), 110.20 (C-5), 112.32 (C-1), 116.23 (C-8), 121.37 (C-7), 124.14 (C-6), 125.20 (C-2), 126.79 (C-2’), 127.07 (C-8a), 127.57 (C-4’), 128.73 (C-3’), 136.25 (C-4a), 136.91 (C-1’), 143.08 (C-3a), 160.68 (COO-1), 163.63 (COO-3) ppm; ^15^N NMR (CDCl_3_) δ 116.9 (N-4), 172.1 (N-9) ppm; anal. calcd. for C_23_H_22_N_2_O_4_ (390.43): C, 70.75; H, 5.68; N, 7.17%; found: C, 70.67; H, 5.61; N, 7.23%.

**General procedure for the reaction of 1-benzylbenzimidazolium bromides (6) with DMAD (3d) in 1,2-epoxybutane.** A mixture of a 1-benzylbenzimidazolium bromide **6** (2 mmol) and DMAD **3d** (2 mmol) in 30 mL of 1,2-epoxybutane was heated at reflux temperature for 24 hours. The solvent was removed under vacuum, and the residue was chromatographed on a SiO_2_ packed column by eluting with EtOAc:hexane (1:4 v/v) giving pyrrolo[1,2-*a*]quinoxalin-4-ones **4h,i** and the pyrrolo[1,2-*a*]benzimidazole **5h** (the order of elution: **4**<**5**).

**Dimethyl 4-oxo-5-benzylpyrrolo[1,2-*a*]quinoxalin-2,3-dicarboxylate (4h).** 0.33 g (42%) white crystals. FTIR (ν_max_, cm^–1^): 1748, 1710, 1663, 1523, 1412, 1370, 1270, 1246, 1198, 1153, 1074; ^1^H NMR (CDCl_3_) δ 3.90 (3H, s, CH_3_-2), 4.05 (3H, s, CH_3_-3), 5.47 (2H, bs, CH_2_), 7.22–7.33 (8H, m, aromatic rings), 7.72–7.74 (1H, m, H-9), 8.19 (1H, s, H-1). The individual chemical shifts, multiplicities and coupling constants for the 7.22–7.33 multiplet were obtained from undecoupled HSQC as follows: 7.24 (1H, t, 7.7 Hz, H-8), 7.25 (1H, t, 7.2 Hz, H-4’), 7.254 (1H, d, 8.7 Hz, H-6), 7.26 (2H, d, 7.9 Hz, H-2’), 7.28 (1H, t, 7.9 Hz, H-7), 7.31 (2H, t, 7.72 Hz, H-3’); ^13^C NMR (CDCl_3_) δ 45.20 (CH_2_), 52.10 (CH_3_-2), 53.12 (CH_3_-3), 115.18 (C-9), 117.07 (C-6), 117.85 (C-2), 118.81 (C-1), 120.80 (C-3a), 121.21 (C-3), 122.66 (C-9a), 123.58 (C-8), 126.58 (C-2’), 127.46 (C-7), 127.55 (C-4’), 128.91 (C-3’), 129.92 (C-5a), 135.57 (C-1’), 154.43 (C-4), 162.84 (COO-2), 165.42 (COO-3) ppm; ^15^N NMR (CDCl_3_) δ 137.6 (N-5), 172.0 (N-10) ppm; anal. calcd for C_22_H_18_N_2_O_5_ (390.39): C, 67.68; H, 4.65; N, 7.18%; found: C, 67.75; H, 4.68; N, 7.12%.

**Dimethyl 1-carbethoxy-4-benzyl-4*H*-pyrrolo[1,2-*a*]benzimidazole-2,3-dicarboxylate (5h).** 0.14 g (16%) pale yellow crystals. FTIR (ν_max_, cm^−1^): 2997, 2951, 1745, 1710, 1687, 1663, 1572, 1522, 1456, 1408, 1369, 1269, 1216, 1177, 1140, 1066, 1074. ^1^H NMR (CDCl_3_) δ 1.44 (3H, t, 7.2 Hz, CH_3_-Et), 3.81 (3H, s, CH_3_-3), 4.01 (3H, s, CH_3_-2), 4.43 (2H, quartet, 7.2 Hz, CH_2_-Et), 6.08 (2H, bs, CH_2_), 7.22–7.38 (8H, m, aromatic rings), 8.86 (1H, d, 8.0 Hz, H-8). The individual chemical shifts, multiplicities and coupling constants for the 7.22–7.38 multiplet were obtained from undecoupled HSQC as follows: 7.22 (2H, d, 7.6 Hz, H-2’), 7.27 (1H, t, 7.5 Hz, H-4’), 7.29 (1H, d, 8.3 Hz, H-5), 7.30 (1H, t, 8.1 Hz, H-7), 7.31 (2H, t, 7.6 Hz, H-3’), 7.35 (1H, t, 8 Hz, H-6) ppm; ^13^C NMR (CDCl_3_) δ 14.21 (CH_3_-Et), 48.51 (CH_2_), 51.58 (CH_3_-3), 52.58 (CH_3_-2), 60.88 (CH_2_-Et), 89.98 (C-3), 109.71 (C-1), 110.29 (C-5), 116.62 (C-8), 121.75 (C-7), 124.79 (C-6), 126.58 (C-2’), 126.63 (C-8a), 127.66 (C-4’), 128.78 (C-3’), 130.49 (C-2), 136.41 (C-4a), 136.57 (C-1), 141.86 (C-3a), 159.58 (COO-Et), 162.76 (COO-3), 166.10 (COO-2) ppm; ^15^N NMR (CDCl_3_) δ 116.1 (N-4), 168.7 (N-9) ppm; anal. calcd for C_24_H_22_N_2_O_6_ (434.44): C, 66.35; H, 5.10; N, 6.45%; found: C, 66.31; H, 5.14; N, 6.39%.

**General synthetic procedure for pyrrolo[1,2-*a*]quinoxalin-4-ones 10a–f.** A mixture of a benzimidazole **9** (2 mmol), alkyl bromoacetate **2** (4 mmol) and a non-symmetrical alkyne **3** (2 mmol) in 30 mL of 1,2-epoxybutane was heated at reflux temperature for 30 hours. The solvent was partly removed under vacuum, 3 mL of MeOH was added under a gentle stirring, and the mixture was left over night in a refrigerator. The formed solid was filtered off and recrystallized from MeOH giving pyrrolo[1,2-*a*]quinoxalin-4-one **10a–f**.

**Ethyl 2-(2-acetyl-4-oxo-pyrrolo[1,2-*a*]quinoxalin-5-yl) acetate (10a).** 0.235 g (38%) beige crystals, mp 193–194 °C. FTIR (ν_max_, cm^−1^): 3109, 2984, 1746, 1656, 1617, 1549, 1516, 1420, 1383, 1357, 1277, 1206; ^1^H NMR (CDCl_3_) δ 1.27 (3H, t, 7.2 Hz, CH_3_-Et), 2.56 (3H, s, CH_3_), 4.25 (2H, quartet, 7.2 Hz, CH_2_-Et), 5.04 (2H, s, CH_2_), 7.08 (1H, d, 8.3 Hz, H-6), 7.28 (1H, t, 7.2 Hz, H-8), 7.36 (1H, t, 7.3 Hz, H-7), 7.59 (1H, d, 1.5 Hz, H-3), 7.75 (1H, d, 8.1 Hz, H-9), 8.21 (1H, d, 1.5 Hz, H-1) ppm; ^13^C NMR (CDCl_3_) δ 14.13 (CH_3_-Et), 27.66 (CH_3_), 42.83 (CH_2_), 61.93 (CH_2_-Et), 113.52 (C-3), 115.39 (C-9), 115.45 (C-6), 118.55 (C-1), 123.19 (C-9a), 123.50 (C-3a), 123.69 (C-8), 127.18 (C-7), 128.48 (C-2), 129.94 (C-5a), 155.07 (C-4), 167.89 (COO), 193.65 (CO)ppm; ^15^N NMR (CDCl_3_) δ 129.9 (N-5), 175.3 (N-10) ppm; anal. calcd for C_17_H_16_N_2_O_4_ (312.32): C, 65.37; H, 5.16; N, 8.97%; found: C, 65.48; H, 5.20; N, 8.88%.

## Supporting Information

File 1 Experimental procedures, characterization data, ^1^H, ^13^C and ^15^N NMR spectra for all new compounds.
