# Supplementary material for: New highlights of the syntheses of pyrrolo[1,2-a]quinoxalin-4-ones
Source: Beilstein J Org Chem. 2014 Oct 14;10:2377–87. doi: 10.3762/bjoc.10.248 (PMC4222434; doi:10.3762/bjoc.10.248)
Supplement: File 1 — Experimental procedures, characterization data, 1H, 13C and 15N NMR spectra for all new compounds. [file Beilstein_J_Org_Chem-10-2377-s001.pdf]

Supporting Information

for

New highlights of the syntheses of pyrrolo[1,2-*a*]quinoxalin-4-ones

Emilian Georgescu[1], Alina Nicolescu[2,3], Florentina Georgescu[4], Florina Teodorescu[2], Daniela Marinescu[1], Ana-Maria Macsim[3] and Calin Deleanu*[2,3]

Address: [1]Research Center Oltchim, Str. Uzinei 1, RO-240050, Ramnicu Valcea, Romania. [2]C. D. Nenitzescu Centre of Organic Chemistry, Romanian Academy, Spl. Independentei 202-B, RO-060023 Bucharest, Romania. [3]Petru Poni Institute of Macromolecular Chemistry, Romanian Academy, Aleea Grigore Ghica Voda 41-A, RO-700487 Iasi, Romania and [4]Research Dept., Teso Spec SRL, Str. Muncii 53, RO-915200 Fundulea, Calarasi, Romania.

Email: Calin Deleanu - calin.deleanu@yahoo.com

*Corresponding author

Experimental procedures, characterization data, ^{1}H, ^{13}C and ^{15}N NMR spectra for all new compounds.

Table of contents

General experimental details.

General procedure and spectral data for **4a–4g, 5b & 5e**

General procedure and spectral data for **4h, 4i & 5h**

General procedure and spectral data for **10a–10f**

General Experimental Details.

Melting points were measured on a Boëtius hot plate microscope and are uncorrected. The IR spectra were recorded on a Nicolet Impact 410 spectrometer, in KBr pellets. The high performance liquid chromatography (HPLC) analyses were performed with an Agilent Chromatograph 1200 Series at room temperature by isocratic elution of acrylonitrile on an Agilent Zorbax SB-C18 (250x4.6) column with flow rate 1.0 mL/min. The NMR spectra have been recorded on a Bruker Avance III 400 instrument operating at 400.1, 100.6 and 40.6 MHz for ^{1}H, ^{13}C, and ^{15}N nuclei respectively. Samples were transferred in 5 mm Wilmad 507 NMR tubes and recorded with either a 5 mm multinuclear inverse detection z-gradient probe (^{1}H spectra and all H-H/H-C/H-N 2D experiments) or with a 5 mm four nuclei direct detection z-gradient probe for ^{13}C spectra. Chemical shifts are reported in δ units (ppm) and were referenced to internal TMS for ^{1}H nuclei, to the internal deuterated solvent for ^{13}C nuclei ($CDCl_3$ referenced at 77.0 ppm), and referenced to liquid ammonia (0.0 ppm) using nitromethane (380.2 ppm) as external standard for ^{15}N nuclei. Unambiguous 1D NMR signal assignments were made based on 2D NMR homo- and heteronuclearcorrelations. H,H-COSY, H,H-NOESY, H,C-HSQC and H,C-HMBC experiments were recorded using standard pulse sequences in the version with z-gradients, as delivered by Bruker with TopSpin 2.1 PL6 spectrometer control and processing software. H,C-undecoupled-HSQC experiments have been recorded using the pulse sequence described by S. Simova [21]. The ^{15}N chemical shifts were obtained as projections from the 2D indirectly detected H,N-HMBC spectra, employing a standard pulse sequence in the version with z-gradients as delivered by Bruker (TopSpin 2.1 PL6). Elemental analyses for C, H and N were obtained using a

COSTECH Instruments EAS32. Satisfactory microanalyses for all new compounds were obtained.

General procedure for the reaction of 1-substituted benzimidazoles (1a-c) with ethyl bromoacetate (2) and non-symmetrical alkynes (3a-c) in 1,2-epoxybutane. A mixture of 1-substituted benzimidazole **1a-c** (2 mmol), ethyl bromoacetate **2** (2 mmol) and an alkyne **3a-c** (2 mmol) in 30 mL of 1,2-epoxybutane was heated at reflux temperature (approx. 62 ºC) for 24 hours. The solvent was partly removed under vacuum, 3 mL of MeOH was added under a gentle stirring, and the mixture was left 2 hours in the refrigerator. The solid formed was filtered off and recrystallized from MeOH/Et_2O giving pyrrolo[1,2-*a*]quinoxalin-4-one **4a-g**. The filtrate was concentrated under vacuum and chromatographed on a SiO_2 packed column by eluting with EtOAc:hexane (1:4 v/v) giving pyrrolo[1,2-*a*]benzimidazole **5** and an additional quantity of pyrrolo[1,2-*a*]quinoxalin-4-one **4** (the order of elution: **4**<**5**).

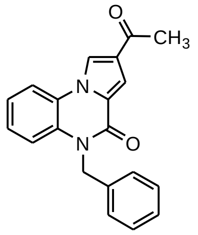

.

2-Acetyl-5-benzylpyrrolo[1,2-*a*]quinoxalin-4-one (4a). 0.246 g (39%) yellow crystals. FT-IR (ν_{max}, cm^{-1}): 3113, 2960, 1650, 1613, 1544, 1515, 1440, 1421, 1355, 1307, 1265, 1207, 1080. ^1H NMR ($CDCl_3$) δ (ppm): 2.58 (3H, s, CH_3), 5.51 (2H, bs, CH_2), 7.20-7.33 (8H, m, aromatic rings), 7.65 (1H, d, 1.6 Hz, H-3), 7.72-7.75 (1H, m, H-9), 8.23 (1H, d, 1.6 Hz, H-1). The individual chemical shifts, multiplicities and coupling constants for the 7.20-7.33 multiplet were obtained from undecoupled HSQC as follows: 7.21-7.25 (1H, m, H-8), 7.253 (1H, d, 8.4 Hz, H-6), 7.256 (1H, t, 7.2 Hz, H-4'), 7.26 (1H, t, 8.2 Hz, H-7), 7.28 (2H, d, 7.4 Hz, H-2'), 7.32 (2H, t, 7.8 Hz, H-3'). ^{13}C NMR ($CDCl_3$) δ (ppm): 27.70 (CH_3), 45.19 (CH_2), 113.32 (C-3), 115.13 (C-

9), 116.98 (C-6), 118.28 (C-1), 123.34 (C-9a), 123.42 (C-8), 123.88 (C-3a), 126.59 (C-2'), 127.08 (C-7), 127.53 (C-4'), 128.50 (C-2), 128.93 (C-3'), 130.13 (C-5a), 135.98 (C-1'), 155.63 (C-4), 193.74 (CO-2). ^{15}N NMR ($CDCl_3$) δ (ppm): 136.1 (N-5), 174.8 (N-10). Anal. Calcd. for $C_{20}H_{16}N_2O_2$ (316.35): C, 75.93; H, 5.10; N, 8.85%. Found: C, 76.01; H, 5.15; N, 8.78%.

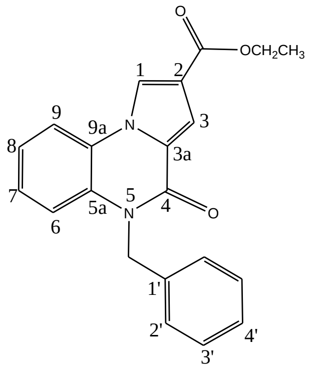

Ethyl 4-oxo-5-benzylpyrrolo[1,2-*a*]quinoxalin-2-carboxylate (4b). 0.29 g (42%) pale yellow crystals. FT-IR (ν_{max}, cm^{-1}): 3121, 2975, 1710, 1651, 1611, 1551, 1519, 1426, 1361, 1305, 1270, 1196, 1165, 1096, 1023. ^{1}H NMR ($CDCl_3$) δ (ppm): 1.41 (3H, t, 7.2 Hz, CH_3), 4.38 (2H, quartet, 7.2 Hz, CH_2), 5.50 (2H, bs, CH_2), 7.19-7.33 (8H, m, aromatic rings), 7.68 (1H, d, 1.6 Hz, H-3), 7.72-7.73 (1H, m, H-9), 8.24 (1H, d, 1.6 Hz, H-1). The individual chemical shifts, multiplicities and coupling constants for the 7.19-7.33 multiplet were obtained from undecoupled HSQC as follows: 7.21 (1H, m, H-8), 7.236 (1H, t, 8.1 Hz, H-7), 7.239 (1H, d, 8.2 Hz, H-6), 7.25 (1H, t, 7.4 Hz, H-4'), 7.28 (2H, d, 7.2 Hz, H-2'), 7.31 (2H, t, 7.3 Hz, H-3'). ^{13}C NMR ($CDCl_3$) δ (ppm): 14.38 (CH_3), 45.12 (CH_2), 60.57 (OCH_2), 113.97 (C-3), 114.99 (C-9), 116.88 (C-6), 119.42 (C-1), 120.43 (C-2), 123.27 (C-8), 123.37 (C-9a), 123.59 (C-3a), 126.58 (C-2'), 126.78 (C-7), 127.45 (C-4'), 128.87 (C-3'), 129.97 (C-5a), 136.04 (C-1'), 155.48 (C-4), 163.77 (COO). ^{15}N NMR ($CDCl_3$) δ (ppm): 136.4 (N-5), 173.5 (N-10). Anal. Calcd. for $C_{21}H_{18}N_2O_3$ (346.38): C, 72.82; H, 5.24; N, 8.09%. Found: C, 72.90; H, 5.31; N, 8.01%.

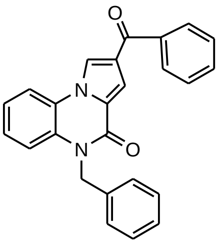

2-Benzoyl-5-benzylpyrrolo[1,2-*a*]quinoxalin-4-one (4c). 0.43 g (57%) white crystals. FT-IR (ν_{max}, cm^{-1}): 3123, 3058, 1657, 1633, 1543, 1517, 1420, 1383, 1305, 1280, 1215, 1178. ^{1}H NMR ($CDCl_3$) δ (ppm): 5.52 (2H, bs, CH_2), 7.21-7.34 (8H, m, aromatic rings), 7.57 (2H, t, 7.7 Hz, H-3”), 7.66 (1H, t, 7.4 Hz, H-4”), 7.72 (1H, d, 1.6 Hz, H-3), 7.80-7.83 (1H, m, H-9), 8.00 (2H, d, 7.0 Hz, H-2”), 8.36 (1H, d, 1.6 Hz, H-1). The individual chemical shifts, multiplicities and coupling constants for the 7.21-7.34 multiplet were obtained from undecoupled HSQC as follows: 7.27 (1H, m, H-8), 7.30 (1H, t, 7.3 Hz, H-4’), 7.311 (1H, t, 8.1 Hz, H-7), 7.313 (1H, d, 8.3 Hz, H-6), 7.34 (2H, d, 7.5 Hz, H-2’), 7.37 (2H, t, 7.6 Hz, H-3’). ^{13}C NMR ($CDCl_3$) δ (ppm): 45.20 (CH_2), 114.96 (C-3), 115.19 (C-9), 116.97 (C-6), 120.20 (C-1), 123.32 (C-9a), 123.41 (C-8), 123.62 (C-3a), 126.58 (C-2’), 127.07 (C-7), 127.22 (C-2), 127.51 (C-4’), 128.50 (C-3”), 128.92 (C-3’), 129.29 (C-2”), 130.09 (C-5a), 132.39 (C-4”), 135.97 (C-1’), 138.33 (C-1”), 155.69 (C-4), 190.46 (CO-2). ^{15}N NMR ($CDCl_3$) δ (ppm): 137.9 (N-5), 175.7 (N-10). Anal. Calcd. for $C_{25}H_{18}N_2O_2$ (378.42): C, 79.35; H, 4.79; N, 7.40%. Found: C, 79.27; H, 4.72; N, 7.51%.

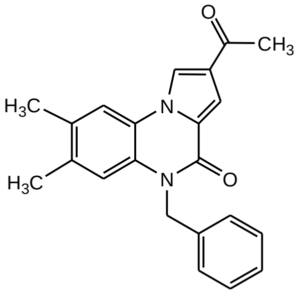

2-Acetyl-5-benzyl-7,8-dimethylpyrrolo[1,2-*a*]quinoxalin-4-one (4d). 0.296 g (43%) yellow crystals. FT-IR (ν_{max}, cm^{-1}): 3113, 2919, 1652, 1625, 1544, 1521, 1416, 1384,

1354, 1280, 1261, 1212. ^1H NMR ($CDCl_3$) δ (ppm): 2.27 (3H, s, CH_3-7), 2.35 (3H, s, CH_3-8), 2.61 (3H, s, CH_3), 5.51 (2H, bs, CH_2), 7.05 (1H, s, H-6), 7.26-7.37 (5H, m, aromatic rings), 7.54 (1H, s, H-9), 7.65 (1H, d, 1.6 Hz, H-3), 8.22 (1H, d, 1.6 Hz, H-1). The individual chemical shifts, multiplicities and coupling constants for the 7.26-7.37 multiplet were obtained from undecoupled HSQC as follows: 7.29 (1H, t, 7.3 Hz, H-4'), 7.31 (2H, d, 7.4 Hz, H-2'), 7.35 (2H, t, 7.4 Hz, H-3'). ^{13}C NMR ($CDCl_3$) δ (ppm): 19.38 (CH_3-8), 20.05 (CH_3-7), 27.64 (CH_3), 45.03 (CH_2), 112.98 (C-3), 115.89 (C-9), 117.67 (C-6), 117.91 (C-1), 121.09 (C-9a), 123.84 (C-3a), 126.60 (C-2'), 127.40 (C-4'), 127.86 (C-5a), 128.17 (C-2), 128.87 (C-3'), 132.24 (C-8), 135.92 (C-7), 136.26 (C-1'), 155.67 (C-4), 193.82 (CO). ^{15}N NMR ($CDCl_3$) δ (ppm): 136.9 (N-5), 176.1 (N-10). Anal. Calcd. for $C_{22}H_{20}N_2O_2$ (344.41): C, 76.72; H, 5.85; N, 8.13%. Found: C, 76.66; H, 5.81; N, 8.20%.

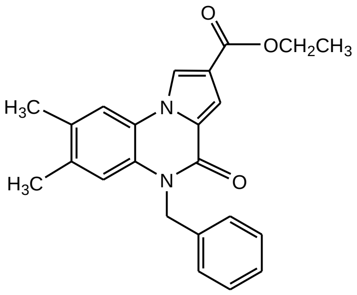

Ethyl 4-oxo-5-benzyl-7,8-dimethylpyrrolo[1,2-*a*]quinoxalin-2-carboxylate (4e). 0.285 g (38%) yellow crystals. FT-IR (ν_{max}, cm^{-1}): 3138, 2980, 1711, 1656, 1597, 1550, 1524, 1418, 1364, 1272, 1255, 1205, 1181, 1096, 1022. ^1H NMR ($CDCl_3$) δ (ppm): 1.40 (3H, t, 7.2 Hz, CH_3-Et), 2.20 (3H, s, CH_3-7), 2.27 (3H, s, CH_3-8), 4.37 (2H, quartet, 7.1 Hz, CH_2-Et), 5.43 (2H, bs, CH_2), 6.96 (1H, s, H-6), 7.21-7.31 (5H, m, aromatic rings), 7.42 (1H, s, H-9), 7.62 (1H, d, 1.6 Hz, H-3), 8.14 (1H, d, 1.6 Hz, H-1). The individual chemical shifts, multiplicities and coupling constants for the 7.21-7.31 multiplet were obtained from undecoupled HSQC as follows: 7.23 (1H, t, 7.3 Hz, H-4'), 7.27 (2H, d, 7.6 Hz, H-2'), 7.30 (2H, t, 7.6 Hz, H-3'). ^{13}C NMR ($CDCl_3$) δ (ppm):

14.43 (CH_3-Et), 19.34 (CH_3-8), 20.00 (CH_3-7), 44.96 (CH_2), 60.49 (CH_2-Et), 113.58 (C-3), 115.72 (C-9), 117.56 (C-6), 119.04 (C-1), 119.99 (C-2), 121.12 (C-9a), 123.58 (C-3a), 126.65 (C-2'), 127.36 (C-4'), 127.67 (C-5a), 128.84 (C-3'), 132.10 (C-8), 135.61 (C-7), 136.36 (C-1'), 155.49 (C-4), 163.90 (COO). ^{15}N NMR ($CDCl_3$) δ (ppm): 136.82 (N-5), 174.91 (N-10). Anal. Calcd. for $C_{23}H_{22}N_2O_3$ (374.43): C, 73.78; H, 5.92; N, 7.48%. Found: C, 73.83; H, 5.97; N, 7.51%.

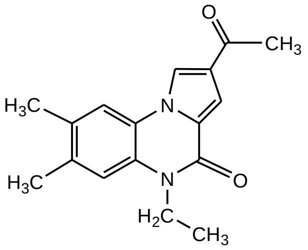

2-Acetyl-5-ethyl-7,8-dimethylpyrrolo[1,2-*a*]quinoxalin-4-one (4f). 0.27 g (48%) beige crystals. FT-IR (ν_{max}, cm^{-1}): 3110, 2971, 2934, 1641, 1624, 1593, 1542, 1519, 1420, 1382, 1359, 1298, 1261, 1224, 1189, 1026. ^{1}H NMR ($CDCl_3$) δ (ppm): 1.35 (3H, t, 7.2 Hz, CH_3-Et), 2.35 (3H, s, CH_3-8), 2.37 (3H, s, CH_3-7), 2.55 (3H, s, CH_3), 4.28 (2H, quartet, 7.2 Hz, CH_2-Et), 7.09 (1H, s, H-6), 7.49 (1H, s, H-9), 7.52 (1H, d, 1.4 Hz, H-3), 8.13 (1H, d, 1.4 Hz, H-1). ^{13}C NMR ($CDCl_3$) δ (ppm): 12.77 (CH_3-Et), 19.39 (CH_3-8), 20.11 (CH_3-7), 27.62 (CH_3), 36.29 (CH_2-Et), 112.24 (C-3), 116.07 (C-9), 116.62 (C-6), 117.53 (C-1), 121.14 (C-9a), 124.10 (C-3a), 127.47 (C-5a), 128.01 (C-2), 131.90 (C-8), 135.97 (C-7), 154.99 (C-4), 193.88 (CO). ^{15}N NMR ($CDCl_3$) δ (ppm): 140.1 (N-5), 175.8 (N-10). Anal. Calcd. for $C_{17}H_{18}N_2O_2$ (282.34): C, 72.32; H, 6.43; N, 9.92%. Found: C, 72.41; H, 6.37; N, 9.83%.

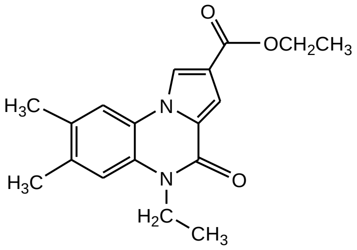

Ethyl 4-oxo-5-ethyl-7,8-dimethylpyrrolo[1,2-*a*]quinoxalin-2-carboxylate (4g). 0.244 g (39%) white crystals. FT-IR (v_{max}, cm^{-1}) 3126, 2978, 2934, 1706, 1655, 1525, 1418, 1364, 1270, 1190, 1088, 1027. ^{1}H NMR ($CDCl_3$) δ (ppm): 1.33 (3H, t, 7.2 Hz, CH_3-Et-N), 1.38 (3H, t, 7.2 Hz, CH_3-Et-O), 2.33 (3H, s, CH_3-8), 2.34 (3H, s, CH_3-7), 4.25 (2H, quartet, 7.2 Hz, CH_2-Et-N), 4.35 (2H, quartet, 7.2 Hz, CH_2-Et-O), 7.05 (1H, s, H-6), 7.44 (1H, s, H-9), 7.53 (1H, d, 1.6 Hz, H-3), 8.10 (1H, d, 1.6 Hz, H-1). ^{13}C NMR ($CDCl_3$) δ (ppm): 12.76 (CH_3-Et-N), 14.37 (CH_3-Et-O), 19.34 (CH_3-8), 20.05 (CH_3-7), 36.20 (CH_2-Et-N), 60.40 (CH_2-Et-O), 112.81 (C-3), 115.87 (C-9), 116.49 (C-6), 118.60 (C-1), 119.75 (C-2), 121.15 (C-9a), 123.80 (C-3a), 127.26 (C-5a), 131.73 (C-8), 135.62 (C-7), 154.80 (C-4), 163.95 (COO). ^{15}N NMR ($CDCl_3$) δ (ppm): 139.8 (N-5), 174.6 (N-10). Anal. Calcd. for $C_{18}H_{20}N_2O_3$ (312.36): C, 69.21; H, 6.45; N, 8.97%. Found: C, 69.15; H, 6.41; N, 8.89%.

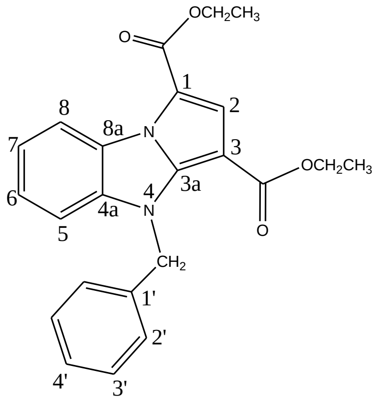

Diethyl 4-benzyl-4*H*-pyrrolo[1,2-*a*]benzimidazole-1,3-dicarboxylate (5b). 0.1g (13%) pale yellow crystals. FT-IR (v_{max}, cm^{-1}): 1700, 1685, 1580, 1514, 1479, 1453, 1400, 1303, 1290, 1233, 1181, 1136, 1106, 1070. ^{1}H NMR ($CDCl_3$) δ (ppm): 1.37 (3H, t, 7.2 Hz, CH_3-3), 1.48 (3H, t, 7.2 Hz, CH_3-1), 4.32(2H, quartet, 7.2 Hz, CH_2-3), 4.45 (2H, quartet, 7.2 Hz, CH_2-1), 6.13 (2H, bs, CH_2), 7.25-7.32 (8H, m, aromatic rings), 7.78 (1H, s, H-2), 8.88 (1H, d, 8.2 Hz, H-8). The individual chemical shifts, multiplicities and coupling constants for the 7.25-7.32 multiplet were obtained from undecoupled HSQC as follows: 7.240 (2H, d, 7.5 Hz, H-2’), 7.248 (1H, t, 7.3 Hz, H-

4'), 7.25 (1H, d, 8.2 Hz, H-5), 7.26 (1H, t, 8 Hz, H-7), 7.29 (2H, t, 7.4 Hz, H-3'), 7.30 (1H, t, 8 Hz, H-6). ^{13}C NMR ($CDCl_3$) δ (ppm): 14.47 (CH_3-3), 14.59 (CH_3-1), 48.48 (CH_2), 59.93 (CH_2-3), 60.24 (CH_2-1), 91.75 (C-3), 110.20 (C-5), 112.32 (C-1), 116.23 (C-8), 121.37 (C-7), 124.14 (C-6), 125.20 (C-2), 126.79 (C-2'), 127.07 (C-8a), 127.57 (C-4'), 128.73 (C-3'), 136.25 (C-4a), 136.91 (C-1'), 143.08 (C-3a), 160.68 (COO-1), 163.63 (COO-3). ^{15}N NMR ($CDCl_3$) δ (ppm): 116.9 (N-4), 172.1 (N-9). Anal. Calcd. for $C_{23}H_{22}N_2O_4$ (390.43): C, 70.75; H, 5.68; N, 7.17%. Found: C, 70.67; H, 5.61; N, 7.23%.

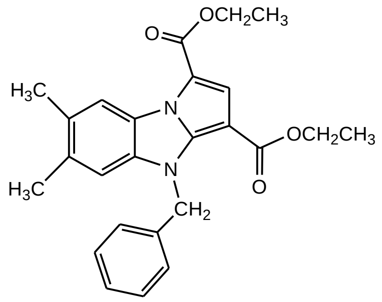

Diethyl 4-benzyl-6,7-dimethyl-4*H*-pyrrolo[1,2-*a*]benzimidazole-1,3-dicarboxylate (5e). 0.175 g (21%) orange crystals. FT-IR (v_{max}, cm^{-1}): 1701, 1675, 1570, 1514, 1452, 1390, 1302, 1230, 1210, 1163, 1152, 1105, 1067. ^{1}H NMR ($CDCl_3$) δ (ppm): 1.31 (3H, t, 7.2 Hz, CH_3-3), 1.43 (3H, t, 7.2 Hz, CH_3-1), 2.30 (3H, s, CH_3-6), 2.37 (3H, s, CH_3-7), 4.25 (2H, quartet, 7.2 Hz, CH_2-3), 4.39 (2H, quartet, 7.2 Hz, CH_2-1), 6.01 (2H, bs, CH_2), 6.97 (1H, s, H-5), 7.18-7.28 (5H, m, aromatic rings), 7.69 (1H, s, H-2), 8.56 (1H, s, H-8). The individual chemical shifts, multiplicities and coupling constants for the 7.18-7.28 multiplet were obtained from undecoupled HSQC as follows: 7.23 (2H, d, 7.1 Hz, H-2'), 7.26 (1H, t, 7.6 Hz, H-4'), 7.30 (2H, t, 7.3 Hz, H-3'). ^{13}C NMR ($CDCl_3$) δ (ppm): 14.47 (CH_3-3), 14.61 (CH_3-1), 20.14 (CH_3-7), 20.43 (CH_3-6), 48.27 (CH_2), 59.82 (CH_2-3), 60.13 (CH_2-1), 91.69 (C-3), 110.60 (C-5), 111.93 (C-1), 116.44 (C-8), 124.88 (C-2), 125.39 (C-8a), 126.64 (C-2'), 127.41 (C-4'), 128.67 (C-3'), 130.20 (C-7), 133.08 (C-6), 134.62 (C-4a), 137.20 (C-1'), 142.92 (C-3a), 160.77

(COO-1), 163.63 (COO-3). ^{15}N NMR ($CDCl_3$) δ (ppm): 115.3 (N-4), 171.4 (N-9). Anal. Calcd. for $C_{25}H_{26}N_2O_4$ (418.48): C, 71.75; H, 6.26; N, 6.69%. Found: C, 71.82; H, 6.31; N, 6.66%.

General procedure for the reaction of 1-benzylbenzimidazolium bromides (6) with DMAD (3d) in 1,2-epoxybutane. A mixture of a 1-benzylbenzimidazolium bromide **6** (2 mmol) and DMAD **3d** (2 mmol) in 30 mL of 1,2-epoxybutane was heated at reflux temperature for 24 hours. The solvent was removed under vacuum, and the residue was chromatographed on a SiO_2 packed column by eluting with EtOAc:hexane (1:4 v/v) giving pyrrolo[1,2-*a*]quinoxalin-4-ones **4h,i** and the pyrrolo[1,2-*a*]benzimidazole **5h** (the order of elution: **4**<**5**).

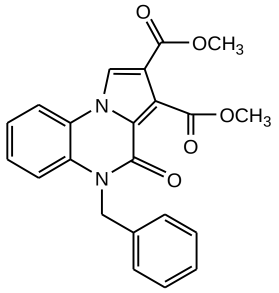

.

Dimethyl 4-oxo-5-benzylpyrrolo[1,2-*a*]quinoxalin-2,3-dicarboxylate (4h). 0.33 g (42%) white crystals. FT-IR (v_{max}, cm^{-1}): 1748, 1710, 1663, 1523, 1412, 1370, 1270, 1246, 1198, 1153, 1074. ^{1}H NMR ($CDCl_3$) δ (ppm): 3.90 (3H, s, CH_3-2), 4.05 (3H, s, CH_3-3), 5.47 (2H, bs, CH_2), 7.22-7.33 (8H, m, aromatic rings), 7.72-7.74 (1H, m, H-9), 8.19 (1H, s, H-1). The individual chemical shifts, multiplicities and coupling constants for the 7.22-7.33 multiplet were obtained from undecoupled HSQC as follows: 7.24 (1H, t, 7.7 Hz, H-8), 7.25 (1H, t, 7.2 Hz, H-4'), 7.254 (1H, d, 8.7 Hz, H-6), 7.26 (2H, d, 7.9 Hz, H-2'), 7.28 (1H, t, 7.9 Hz, H-7), 7.31 (2H, t, 7.72 Hz, H-3'). ^{13}C NMR ($CDCl_3$) δ (ppm): 45.20 (CH_2), 52.10 (CH_3-2), 53.12 (CH_3-3), 115.18 (C-9), 117.07 (C-6), 117.85 (C-2), 118.81 (C-1), 120.80 (C-3a), 121.21 (C-3), 122.66 (C-

9a), 123.58 (C-8), 126.58 (C-2'), 127.46 (C-7), 127.55 (C-4'), 128.91 (C-3'), 129.92 (C-5a), 135.57 (C-1'), 154.43 (C-4), 162.84 (COO-2), 165.42 (COO-3). ^{15}N NMR ($CDCl_3$) δ (ppm): 137.6 (N-5), 172.0 (N-10). Anal. Calcd. for $C_{22}H_{18}N_2O_5$ (390.39): C, 67.68; H, 4.65; N, 7.18%. Found: C, 67.75; H, 4.68; N, 7.12%.

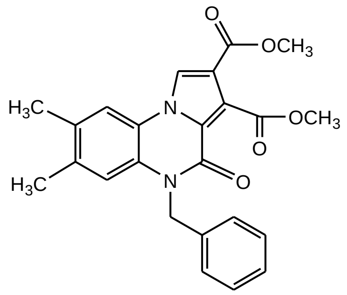

Dimethyl 4-oxo-5-benzyl-7,8-dimethylpyrrolo[1,2-*a*]quinoxalin-2,3-dicarboxylate (4i). 0.16 g (19%) yellow crystals. FT-IR (v_{max}, cm^{-1}): 2950, 1736, 1721, 1657, 1560, 1522, 1441, 1403, 1376, 1324, 1274, 1250, 1222, 1200, 1072. ^{1}H NMR ($CDCl_3$) δ (ppm): 2.22 (3H, s, CH_3-7), 2.31 (3H, s, CH_3-8), 3.89 (3H, s, CH_3-2), 4.04 (3H, s, CH_3-3), 5.43 (2H, s, CH_2), 7.01 (1H, s, H-6), 7.24-7.30 (5H, m, aromatic rings), 7.48 (1H, s, H-9), 8.14 (1H, s, H-1). The individual chemical shifts, multiplicities and coupling constants for the 7.24-7.30 multiplet were obtained from undecoupled HSQC as follows: 7.24 (1H, t, 7.3 Hz, H-4'), 7.25 (2H, d, 7.7 Hz, H-2'), 7.30 (2H, t, 7.5 Hz, H-3'). ^{13}C NMR ($CDCl_3$) δ (ppm): 19.38 (CH_3-8), 20.06 (CH_3-7), 45.08 (CH_2), 52.02 (CH_3-2), 53.08 (CH_3-3), 115.91 (C-9), 117.48 (C-2), 117.78 (C-6), 118.46 (C-1), 120.46 (C-9a and C-3a), 120.86 (C-3), 126.65 (C-2'), 127.45 (C-4'), 127.73 (C-5a), 128.87 (C-3'), 132.50 (C-8), 135.91 (C-1'), 136.42 (C-7), 154.47 (C-4), 162.98 (COO-2), 165.61 (COO-3). ^{15}N NMR ($CDCl_3$) δ (ppm): 137.7 (N-5), 173.3 (N-10). Anal. Calcd. for $C_{24}H_{22}N_2O_5$ (418.44): C, 68.89; H, 5.30; N, 6.69%. Found: C, 68.92; H, 5.36; N, 6.62%.

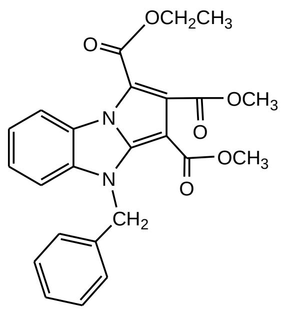

Dimethyl 1-carbethoxy-4-benzyl-4*H*-pyrrolo[1,2-*a*]benzimidazole-2,3-dicarboxylate (5h). 0.14 g (16%) pale yellow crystals. FT-IR (ν_{max}, cm^{-1}): 2997, 2951, 1745, 1710, 1687, 1663, 1572, 1522, 1456, 1408, 1369, 1269, 1216, 1177, 1140, 1066, 1074. ^{1}H NMR ($CDCl_3$) δ (ppm): 1.44 (3H, t, 7.2 Hz, CH_3-Et), 3.81 (3H, s, CH_3-3), 4.01 (3H, s, CH_3-2), 4.43 (2H, quartet, 7.2 Hz, CH_2-Et), 6.08 (2H, bs, CH_2), 7.22-7.38 (8H, m, aromatic rings), 8.86 (1H, d, 8.0 Hz, H-8). The individual chemical shifts, multiplicities and coupling constants for the 7.22-7.38 multiplet were obtained from undecoupled HSQC as follows: 7.22 (2H, d, 7.6 Hz, H-2'), 7.27 (1H, t, 7.5 Hz, H-4'), 7.29 (1H, d, 8.3 Hz, H-5), 7.30 (1H, t, 8.1 Hz, H-7), 7.31 (2H, t, 7.6 Hz, H-3'), 7.35 (1H, t, 8 Hz, H-6). ^{13}C NMR ($CDCl_3$) δ (ppm): 14.21 (CH_3-Et), 48.51 (CH_2), 51.58 (CH_3-3), 52.58 (CH_3-2), 60.88 (CH_2-Et), 89.98 (C-3), 109.71 (C-1), 110.29 (C-5), 116.62 (C-8), 121.75 (C-7), 124.79 (C-6), 126.58 (C-2'), 126.63 (C-8a), 127.66 (C-4'), 128.78 (C-3'), 130.49 (C-2), 136.41 (C-4a), 136.57 (C-1), 141.86 (C-3a), 159.58 (COO-Et), 162.76 (COO-3), 166.10 (COO-2). ^{15}N NMR ($CDCl_3$) δ (ppm): 116.1 (N-4), 168.7 (N-9). Anal. Calcd. for $C_{24}H_{22}N_2O_6$ (434.44): C, 66.35; H, 5.10; N, 6.45%. Found: C, 66.31; H, 5.14; N, 6.39%.

General synthetic procedure for pyrrolo[1,2-*a*]quinoxalin-4-ones 10a-f. A mixture of a benzimidazole **9** (2 mmol), alkyl bromoacetate **2** (4 mmol) and a non-symmetrical alkyne **3** (2 mmol) in 30 mL of 1,2-epoxybutane was heated at reflux temperature for 30 hours. The solvent was partly removed under vacuum, 3 mL of MeOH was added

under a gentle stirring, and the mixture was left over night in the refrigerator. The formed solid was filtered off and recrystallized from MeOH giving pyrrolo[1,2-*a*]quinoxalin-4-one **10a-f**.

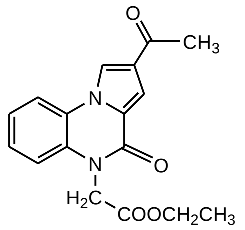

Ethyl 2-(2-acetyl-4-oxo-pyrrolo[1,2-*a*]quinoxalin-5-yl) acetate (10a). 0.235 g (38%) beige crystals, mp 193-194 °C. FT-IR (ν_{max}, cm^{-1}): 3109, 2984, 1746, 1656, 1617, 1549, 1516, 1420, 1383, 1357, 1277, 1206. ^{1}H NMR ($CDCl_3$) δ (ppm): 1.27 (3H, t, 7.2 Hz, CH_3-Et), 2.56 (3H, s, CH_3), 4.25 (2H, quartet, 7.2 Hz, CH_2-Et), 5.04 (2H, s, CH_2), 7.08 (1H, d, 8.3 Hz, H-6), 7.28 (1H, t, 7.2 Hz, H-8), 7.36 (1H, t, 7.3 Hz, H-7), 7.59 (1H, d, 1.5 Hz, H-3), 7.75 (1H, d, 8.1 Hz, H-9), 8.21 (1H, d, 1.5 Hz, H-1). ^{13}C NMR ($CDCl_3$) δ (ppm): 14.13 (CH_3-Et), 27.66 (CH_3), 42.83 (CH_2), 61.93 (CH_2-Et), 113.52 (C-3), 115.39 (C-9), 115.45 (C-6), 118.55 (C-1), 123.19 (C-9a), 123.50 (C-3a), 123.69 (C-8), 127.18 (C-7), 128.48 (C-2), 129.94 (C-5a), 155.07 (C-4), 167.89 (COO), 193.65 (CO). ^{15}N NMR ($CDCl_3$) δ (ppm): 129.9 (N-5), 175.3 (N-10). Anal. Calcd. for $C_{17}H_{16}N_2O_4$ (312.32): C, 65.37; H, 5.16; N, 8.97%. Found: C, 65.48; H, 5.20; N, 8.88%.

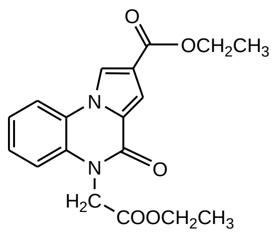

Ethyl 2-(2-carbethoxy-4-oxo-pyrrolo[1,2-*a*]quinoxalin-5-yl) acetate (10b). 0.27 g (40%) yellow crystals, mp 213-214 °C. FT-IR (ν_{max}, cm^{-1}): 3131, 2985, 1737, 1709, 1665, 1555, 1522, 1424, 1391, 1360, 1305, 1280, 1207, 1100, 1021. ^{1}H NMR

($CDCl_3$) δ (ppm): 1.28 (3H, t, 7.1 Hz, CH_3-5), 1.41 (3H, t, 7.1 Hz, CH_3-2), 4.26 (2H, quartet, 7.1 Hz, CH_2-5), 4.38 (2H, quartet, 7.1 Hz, CH_2-2), 5.05 (2H, s, CH_2), 7.08 (1H, d, 8.1 Hz, H-6), 7.29 (1H, t, 7.6 Hz, H-8), 7.37 (1H, t, 7.4 Hz, H-7), 7.64 (1H, d, 1.0 Hz, H-3), 7.76 (1H, d, 7.9 Hz, H-9), 8.24 (1H, d, 1.0 Hz, H-1). ^{13}C NMR ($CDCl_3$) δ (ppm): 14.14 (CH_3-5), 14.38 (CH_3-2), 42.84 (CH_2), 60.61 (CH_2-2), 61.91 (CH_2-5), 114.27 (C-3), 115.31 (C-9), 115.42 (C-6), 119.71 (C-1), 120.47 (C-2), 123.31 (C3a and C-9a), 123.59 (C-8), 126.94 (C-7), 129.88 (C-5a), 154.99 (C-4), 163.71 (COO-2), 167.96 (COO-5). ^{15}N NMR ($CDCl_3$) δ (ppm): 130.2 (N-5), 174.4 (N-10). Anal. Calcd. for $C_{18}H_{18}N_2O_5$ (342.35): C, 63.15; H, 5.30; N, 8.18%. Found: C, 63.09; H, 5.24; N, 8.21%.

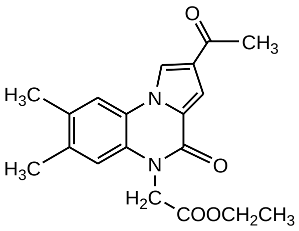

Ethyl 2-(2-acetyl-4-oxo-7,8-dimethylpyrrolo[1,2-*a*]quinoxalin-5-yl) acetate (10c). 0.22 g (32%) pale yellow crystals, mp 274-276 °C. FT-IR (ν_{max}, cm^{-1}): 3119, 2994, 2915, 1741, 1676, 1649, 1549, 1521, 1417, 1357, 1285, 1211, 1098. ^{1}H NMR ($CDCl_3$ +TFA) δ (ppm): 1.31 (3H, t, 7.2 Hz, CH_3-Et), 2.36 (3H, s, CH_3-7), 2.37 (3H, s, CH_3-8), 2.67 (3H, s, C$\underline{H_3}$CO), 4.31 (2H, quartet, 7.2 Hz, CH_2-Et), 5.10 (2H, s, CH_2), 6.92 (1H, s, H-6), 7.61 (1H, s, H-9), 7.73 (1H, d, 1.2 Hz, H-3), 8.41 (1H, d, 1.6 Hz, H-1). ^{13}C NMR ($CDCl_3$ +TFA) δ (ppm): 13.92 (CH_3-Et), 19.44 (CH_3-8), 20.10 (CH_3-7), 27.14 ($\underline{C}H_3$CO), 43.60 (CH_2), 63.00 (CH_2-Et), 115.07 (C-3), 116.52 (C-9), 116.60 (C-6), 120.31 (C-1), 120.97 (C-9a), 122.68 (C-3a), 126.60 (C-5a), 127.44 (C-2), 134.56 (C-8), 137.47 (C-7), 156.63 (C-4), 168.75 (COO), 198.48 (CO). ^{15}N NMR ($CDCl_3$ +TFA) δ (ppm): 131.7 (N-5), 177.9 (N-10). Anal. Calcd. for $C_{19}H_{20}N_2O_4$ (340.37): C, 67.05; H, 5.92; N, 8.234%. Found: C, 67.13; H, 5.99; N, 8.17%.

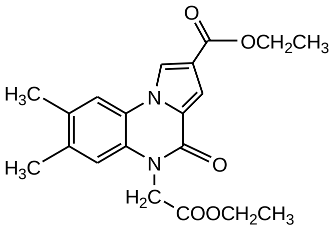

Ethyl 2-(2-carbethoxy-4-oxo-7,8-dimethylpyrrolo[1,2-*a*]quinoxalin-5-yl) acetate (10d). 0.28 g (38%) yellow crystals, mp 214-216 °C. FT-IR (ν_{max}, cm^{-1}): 3121, 2976, 2903, 1741, 1710, 1661, 1527, 1471, 1419, 1363, 1285, 1265, 1205, 1190, 1105, 1028. ^{1}H NMR ($CDCl_3$) δ (ppm): 1.28 (3H, t, 7.2 Hz, CH_3-Et-5), 1.39 (3H, t, 7.2 Hz, CH_3-Et-2), 2.28 (3H, s, CH_3-7), 2.30 (3H, s, CH_3-8), 4.26 (2H, quartet, 7.2 Hz, CH_2-Et-5), 4.35 (2H, quartet, 7.2 Hz, CH_2-Et-2), 4.99 (2H, s, CH_2), 6.77 (1H, s, H-6), 7.26 (1H, s, H-9), 7.57 (1H, d, 1.2 Hz, H-3), 8.12 (1H, d, 1.2 Hz, H-1). ^{13}C NMR ($CDCl_3$) δ (ppm): 14.14 (CH_3-Et-5), 14.39 (CH_3-Et-2), 19.33 (CH_3-8), 19.99 (CH_3-7), 42.71 (CH_2), 60.45 (CH_2-Et-2), 61.80 (CH_2-Et-5), 113.78 (C-3), 116.00 (C-9), 116.06 (C-6), 119.36 (C-1), 119.92 (C-2), 120.99 (C-9a), 123.20 (C-3a), 127.46 (C-5a), 132.37 (C-8), 135.76 (C-7), 154.99 (C-4), 163.79 (COO-2), 168.16 (COO-5). ^{15}N NMR ($CDCl_3$) δ (ppm): 128.9 (N-5), 174.5 (N-10). Anal. Calcd. for $C_{20}H_{22}N_2O_5$ (370.40): C, 64.85; H, 5.99; N, 7.56%. Found: C, 64.91; H, 6.03; N, 7.49%.

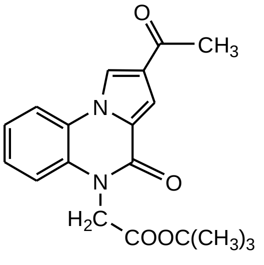

***tert*-Butyl 2-(2-acetyl-4-oxo-pyrrolo[1,2-*a*]quinoxalin-5-yl) acetate (10e).** 0.21 g (31%), pale yellow crystals, mp 197-199 °C. FT-IR (ν_{max}, cm^{-1}): 3133, 3113, 2975, 1742, 1660, 1616, 1549, 1517, 1410, 1362, 1277, 1232, 1216, 1153. ^{1}H NMR ($CDCl_3$) δ (ppm): 1.47 (9H, s, CH_3), 2.56 (3H, s, CH_3-CO), 4.95 (2H, s, CH_2), 7.08 (1H, d, 8.4 Hz, H-6), 7.29 (1H, t, 8.4 Hz, H-8), 7.38 (1H, t, 8.4 Hz, H-7), 7.60 (1H, d,

1.6 Hz, H-3), 7.76 (1H, d, 8 Hz, H-9), 8.21 (1H, d, 1.6 Hz, H-1). ^{13}C NMR ($CDCl_3$) δ (ppm): 27.68 (CH_3-CO), 28.00 (CH_3), 43.49 (CH_2), 113.48 (C-3), 115.37 (CH-9), 115.52 (CH-6), 118.45 (CH-1), 123.19 (C9a), 123.611 (CH-8 and C-3a), 127.12 (CH-7), 128.42 (C-2), 130.08 (C-5a), 155.09 (C-4), 166.92 (COO), 193.72 (CO). ^{15}N NMR ($CDCl_3$) δ (ppm): 130.9 (N-5), 175.2 (N-10). Anal. Calcd. for $C_{19}H_{20}N_2O_4$ (340.37): C, 67.04; H, 5.92; N, 8.23%. Found: C, 67.15; H, 6.02; N, 8.17%.

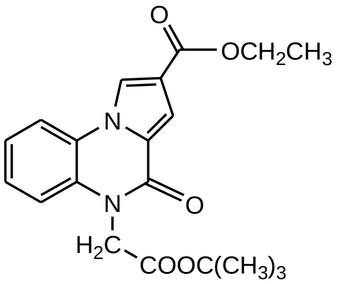

***tert*-Butyl 2-(2-carbethoxy-4-oxo-pyrrolo[1,2-*a*]quinoxalin-5-yl) acetate (10f).** 0.24 g (32%), yellow crystals, mp 159-161°C. FT-IR (ν_{max}, cm^{-1}): 3132, 2984, 1742, 1707, 1665, 1556, 1525, 1380, 1279, 1229, 1213, 1157. ^{1}H NMR ($CDCl_3$) δ (ppm): 1.40 (3H, t, 7.2 Hz, CH_3-Et), 1.46 (9H, s, CH_3), 4.36 (2H, quartet,7.2 Hz, CH_2-Et), 4.94 (2H, s, CH_2), 7.07 (1H, d, 8.4 Hz, H-6), 7.27 (1H, t, 8.4 Hz, H-8), 7.35 (1H, t, 8.4 Hz, H-7), 7.62 (1H, d, 1.6 Hz, H-3), 7.75 (1H, d, 8 Hz, H-9), 8.22 (1H, d, 1.6 Hz, H-1). ^{13}C NMR ($CDCl_3$) δ (ppm): 14.37 (CH_3-Et), 27.99 (CH3), 43.45 (CH_2), 60.58 (CH_2-Et), 114.16 (C-3), 115.25 (CH-9), 115.45 (CH-6), 119.61 (CH-1), 120.39 (C-2), 123.25 (C9a), 123.34 (C-3a), 123.48 (CH-8), 126.84 (CH-7), 129.45 (C-5a), 154.96 (C-4), 163.74 (COO-Et), 166.99 (COO). ^{15}N NMR ($CDCl_3$) δ (ppm): 130.9 (N-5), 174.0 (N-10). Anal. Calcd. for $C_{20}H_{22}N_2O_5$ (370.40): C, 64.85; H, 5.99; N, 7.56%. Found: C, 64.92; H, 6.08; N, 7.48%.
